# Dosing Interval Extension of Dupilumab in CRSwNP: Five‐Year Real World Outcomes

**DOI:** 10.1002/alr.70125

**Published:** 2026-03-02

**Authors:** Nicholas J. Campion, Dioni‐Pinelopi Petsiou, Florian C. Fally, Karina Berbalk, Noah F. Melamed, Aldine Tu, Christina Morgenstern, Fana Alem Kidane, Mohammed Zghaebi, Linda Liu, Minghao Pan, Tina J. Bartosik, Victoria Stanek, Katarina Gangl, Julia Eckl‐Dorna, Sven Schneider

**Affiliations:** ^1^ Vienna Airway Lab Department of Otorhinolaryngology General Hospital and Medical University of Vienna Vienna Austria

**Keywords:** biologics, CRS, CRSwNP, drug safety, dupilumab, long‐term disease outcomes, nasal polyps, real world, sinusitis

## Abstract

**Background:**

Chronic rhinosinusitis with nasal polyps (CRSwNP) is a persistent, often Type 2‐mediated inflammatory disease that markedly impairs quality of life. While dupilumab provides rapid improvement, there is limited evidence on long‐term outcomes beyond 2 years, and the clinical impact of dosing‐interval extension remains unclear. We therefore set out to evaluate long‐term real‐world outcomes of dupilumab therapy in CRSwNP and assess the effectiveness and safety of dosing‐interval extension after achieving disease control.

**Methods:**

This retrospective single‐center cohort included 224 adults with CRSwNP (37% with nonsteroidal anti‐inflammatory drug‐exacerbated respiratory disease) treated with dupilumab for up to 4.5 years with outcomes modeled to 5 years. Longitudinal changes in polyp size, symptom burden, olfaction, asthma control, and Type 2 biomarkers were analyzed using mixed‐effects models. Outcomes were then compared between patients who maintained standard 2‐week dosing and those who voluntarily extended dosing intervals after achieving stable control.

**Results:**

Dupilumab led to significant improvements in polyp burden, olfactory function, and quality of life peaking within 6 months, with sustained benefit through 5 years according to longitudinal modeling. Forty percent of patients extended dosing intervals without loss of efficacy and reported fewer treatment‐related adverse events. Overall, 16% experienced side effects, most commonly musculoskeletal complaints, followed by skin reactions and injection site reactions.

**Conclusion:**

Long‐term dupilumab therapy provided durable disease control and excellent safety. Personalized dosing‐interval extension maintained efficacy and reduced treatment burden, supporting its potential role in optimizing long‐term management of CRSwNP, especially in patients with troublesome side effects.

**Abbreviations**
ACT: asthma control test
AE: adverse event
AEC: absolute eosinophil count
AQLQ: Asthma Quality of Life Questionnaire
CRS: chronic rhinosinusitis
CRSwNP: chronic rhinosinusitis with nasal polyps
ECP: eosinophil cationic protein
ENT: ear, nose, throat
EPOS: European Position Paper on Rhinosinusitis and Nasal Polyps
EMA: European Medicines Agency
Ig: immunoglobin
IL: interleukin
IQR: interquartile range
N‐ERD: NSAID exacerbated respiratory disease
NSAID: nonsteroidal anti‐inflammatory drugs
PROM: patient‐reported outcome measure
QoL: quality of life
SNOT‐22: 22 Item sinonasal outcome test
TPS: total polyp score
VAS: visual analogue score

## 1 Background

Chronic rhinosinusitis with nasal polyps (CRSwNP) is a distinct form of chronic rhinosinusitis (CRS), with an estimated prevalence of 0.65–2% in the Western world [1, 2]. It is characterized by persistent inflammation of the paranasal sinuses, causing symptoms such as nasal congestion, facial pressure, and olfactory dysfunction, which substantially impair quality of life (QoL) and contribute to a considerable burden on healthcare and the economy [3, 4]. CRSwNP is often driven by Type 2 inflammation mediated by interleukin (IL)‐4, IL‐5, and IL‐13 [5]. Dupilumab, a fully human monoclonal antibody that inhibits IL‐4 and IL‐13 signaling, has redefined the treatment landscape for severe, uncontrolled CRSwNP. It is now a widely used and highly effective approved biologic for this indication, endorsed by both European Position Paper on Rhinosinusitis and Nasal Polyps (EPOS) and European Medicines Agency (EMA) guidelines as an add‐on therapy for patients with persistent disease despite standard care [6, 7, 8]. The pivotal SINUS‐24 and SINUS‐52 Phase 3 trials, together with real‐world evidence, have confirmed its short‐term safety and efficacy in improving nasal symptoms and QoL [9, 10]. However, questions remain regarding its sustained performance in everyday practice. Long‐term data are limited, with two clinical follow‐up studies up to 3 years [11, 12], and only one, but very small real‐world cohort extending to 5 years from Israel [13]. Furthermore, data on the impact of patient‐driven treatment adaptations such as interval extension on treatment outcomes and safety remain limited [14, 15].

To address these gaps, particularly the lack of long‐term real‐world evidence from western populations, we evaluated the long‐term effectiveness and safety of dupilumab under both standard and extended dosing intervals in a real‐world cohort of 224 patients with CRSwNP followed for up to 5 years. Thirty‐seven percent of patients had comorbid nonsteroidal anti‐inflammatory drug (NSAID) exacerbated respiratory disease (N‐ERD). We characterized longitudinal treatment trajectories across multiple domains, including nasal polyp burden, patient‐reported outcomes, olfactory function, and Type 2 inflammatory biomarkers. In addition, we evaluated baseline predictors of dupilumab treatment failure and investigated the clinical and safety consequences of patient‐driven dosing interval extension, including factors associated with successful dosing extension.

## 2 Methods

### 2.1 Study Conduct

This retrospective, single‐center cohort study was conducted at the Department of Otorhinolaryngology, Medical University of Vienna, General Hospital of Vienna, Austria. Ethical approval was obtained from the institutional review board (EK: 1340/2025). Data were collected from patients treated between November 2019 and May 2025.

### 2.2 Study Population and Procedures

A total of 224 patients with CRSwNP, diagnosed according to EPOS 2020 criteria, were included [6]. All patients received dupilumab in accordance with EMA prescribing guidelines [16] and had at least one baseline and one follow‐up visit. Total time of follow‐up was up to 4 years, 6 months, and 20 days postinitiation. The number of patients with at least one follow‐up visit in each posttreatment year was: 224 patients in year 0–1, 118 in year 1–2, 67 in year 2–3, 56 in year 3–4, and 15 in year 4–5. Follow‐up intervals for individual patients varied according to clinical practice but typically included baseline, 3, 6, and 12 months posttherapy begin and then annually.

At each visit, demographic and clinical data were recorded, including sex, age, asthma, allergy, N‐ERD, smoking status, and prior surgical history. Blood samples were collected at each visit to assess eosinophilic cationic protein (ECP), total immunoglobin (Ig)E, and absolute eosinophil count (AEC). Nasal polyp burden was graded using the total polyp score (TPS) [17], and smell function was assessed via the validated German Sniffin’ sticks 16 identification test (SSIT‐16) (Burghart Messtechnik GmbH, Holm, Germany). Patient‐reported outcome measures (PROMs) included the 22 item sinonasal outcome test (SNOT‐22), asthma control test (ACT), asthma QoL questionnaire (AQLQ), and a 10‐cm visual analogue scale (VAS) for respiratory allergy burden.

### 2.3 Data Management

All data were anonymized and securely stored on password‐protected hospital servers by members of the Vienna Airway Lab research group (AT, FCT, NFM).

### 2.4 Statistical Analysis and Modeling

For descriptive statistics, categorical data are presented as percentages and continuous data are presented as median and interquartile range [IQR] as no continuous variables were normally distributed according to Shapiro–Wilk testing.

Due to variable follow‐up times among patients, longitudinal outcomes after dupilumab were analyzed using linear mixed‐effects models with a natural cubic spline for time (3 degrees of freedom) to capture the rapid early improvement and subsequent plateau. Time was expressed in months from treatment start. Each model included a patient‐specific random intercept and random slope for time to account for repeated measures and differing rates of change. For each outcome, 𝑌_𝑖𝑗_ measured for patient 𝑖 at time 𝑗 (in months since dupilumab initiation), the following linear mixed‐effects model with a natural cubic spline for time was fitted: 𝑌_𝑖𝑗_ = 𝛽0+(𝑡_𝑖𝑗_) + 𝑏0_𝑖 _+ 𝜀_𝑖𝑗_. Patients who were lost to follow‐up or who discontinued dupilumab due to insufficient clinical response were not excluded from the overall analyses; all available longitudinal data up to the point of discontinuation or loss to follow‐up were retained and included where applicable. Analyses were performed in R (v4.5.1; R Core Team, R Foundation for Statistical Computing, Vienna, Austria; https://www.R‐project.org) using packages stats, lme4 v1.1‐37, lmerTest v3.1‐3, splines v4.5.1 (base), emmeans v1.11.2, and ggplot2 v4.0.0.

Patients who discontinued dupilumab due to insufficient clinical response were identified based on documented clinician‐reported reasons at the final recorded visit. Patients who discontinued for nonefficacy‐related reasons, adverse events (AEs), or who were lost to follow‐up were excluded from analyses of treatment failure.

For interval dosing comparisons, patients were stratified into standard (consistent 2‐week dosing) and extended (>2 week) injection intervals. Outcomes were summarized by 12‐month bins (0–12, 12–24, 24–36, 36–48, 48–60 months) using median [IQR]. Mann–Whitney *U* tests were used for group comparisons, and false discovery rate correction using Benjamini–Hochberg was applied. Baseline predictors of dosing interval extension were assessed using univariable analyses (Fisher's exact test for categorical variables) and multivariable logistic regression, with dosing interval extension (>2 weeks) as the binary outcome. Odds ratios (ORs) with 95% confidence intervals (CIs) are reported. To evaluate predictors of durable dosing interval extension, sensitivity analyses were performed excluding patients who initially extended dosing but later required interval reduction.

## 3 Results

### 3.1 Overview of Study Population

A total of 224 patients receiving dupilumab for CRSwNP were included. The median age was 48 years, and 57% of patients were male. Active smoking was reported by 14% of the cohort. The median duration of disease prior to treatment was 10.6 years, and the median number of previous sinus surgeries was one. Asthma, environmental allergies, and N‐ERD were present in 66%, 55%, and 37% of patients, respectively. The median treatment duration across the cohort was 21 months, ranging from 0.4 to 55 months (Table 1).

**TABLE 1:** Baseline characteristics of patients with CRSwNP treated with dupilumab.

Characteristic	Value
Age (years)	48 (15–82)
Sex	M: 57.1% (128); F: 42.9% (96)
Previous surgeries	1 (0–10)
Smokers	13.8% (31)
Asthma	65.6% (147)
Allergy	54.5% (122)
N‐ERD	36.6% (82)
Time on dupilumab (months)	20.7 (0.4–54.6)
Time since diagnosis (years)	10.6 (0.4–54.2)

*Note*: Values are presented as median (range) or percentage (*n*) as appropriate. Percentages are based on the total number of patients at baseline (V0).

Abbreviation: N‐ERD, nonsteroidal anti‐inflammatory drug exacerbated respiratory disease.

[Correction added on May 09, 2026 after first online publication. Corrections have been made to Table 1, Results, and supplemental tables as specified in the erratum, alr.70172.]

### 3.2 Dupilumab Rapidly Improves Polyp Burden, Smell, and PROMs in the First 6 Months, Stabilizing Thereafter

**FIGURE 1:**
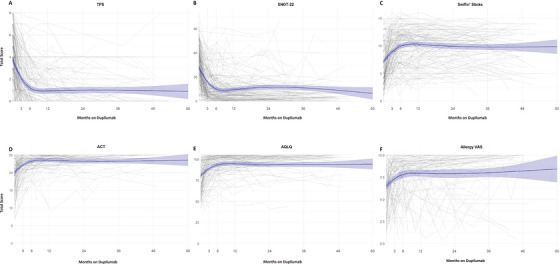
Longitudinal outcomes during dupilumab therapy for patients with CRSwNP. Model predicted population trajectories (blue) with 95% CI (shaded) are shown for (A) total polyp score (TPS), (B) sinonasal outcome test 22 (SNOT‐22), (C) Sniffin’ sticks 16 identity test, (D) asthma control test (ACT), (E) asthma quality of life questionnaire (AQLQ), and (F) burden of allergic symptoms represented by allergic visual analogue scale (VAS). Gray lines denote individual trajectories for clinical outcomes (A–F). Time is months since dupilumab initiation.

In the model, significant improvements in clinical outcomes were observed during the first 6 months of dupilumab therapy, with most measures stabilizing thereafter (Figure 1A–F). Nasal polyp burden, measured by TPS, declined by 1.67 points at 3 months and 2.54 points at 6 months, with minimal further change thereafter, averaging approximately 0.01 points per 100 days. Similarly, the SNOT‐22 score showed a substantial decrease of 17.81 points at 3 months and 26.76 points at 6 months, followed by a continued but modest improvement of 0.34 points per 100 days. Olfactory function improved as well, with Sniffin’ sticks scores increasing by 1.79 and 2.82 points at 3 and 6 months, respectively, before reaching a plateau, with very minimal change thereafter (average of −0.01 points per 100 days). Asthma‐related QoL, as measured by the AQLQ, improved by 8.43 points at 3 months and 12.90 points at 6 months, with minimal additional change thereafter (−0.03 per 100 days). The ACT score followed a similar trajectory, increasing by 2.11 and 3.21 points at 3 and 6 months, respectively, with negligible change after that (0.0001 per 100 days). Respiratory allergy burden, measured by allergy VAS, improved by 0.86 and 1.33 points at 3 and 6 months, with a continued gradual improvement of 0.04 points per 100 days beyond that.

**FIGURE 2:**
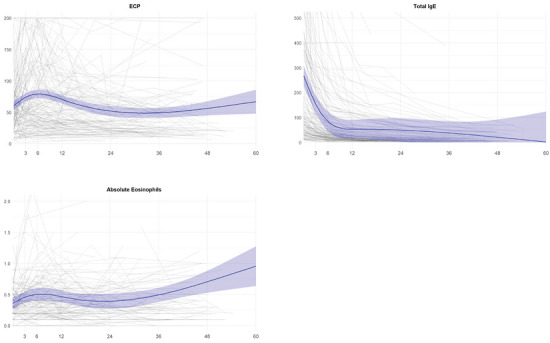
Longitudinal outcomes during dupilumab therapy for patients with CRSwNP. Population trajectories (blue) with 95% CI (shaded) are shown for (A) eosinophil cationic protein (ECP), (B) total IgE, and (C) absolute eosinophils. Gray “spaghetti” lines denote individual trajectories for clinical outcomes (A–C). Time is months since dupilumab initiation.

Biomarker responses varied in their direction and magnitude. ECP (µg/L) levels increased by 13.39 µg/L at 3 months and 18.46 µg/L at 6 months, followed by a gradual decline at a rate of −0.32 µg/L per 100 days. Total IgE (kU/L) levels showed a strong initial decrease of 114.92 kU/L at 3 months and 182.97 kU/L at 6 months, continuing to decline thereafter by approximately −3.87 kU/L per 100 days. In contrast, AEC (×10^9^ cells/L) increased slightly over time, rising by 0.10 × 10^9^ and 0.15 × 10^9^ cells/L at 3 and 6 months, respectively. Across the remainder of the time course, there was a net positive increase at a rate of 0.04 × 10^9^ cells/L per 100 days (Figure 2A–C). All modeled trajectories were statistically significant.

### 3.3 AEs and Medication Tolerability

AEs were reported in 16% of patients and occurred in 6% of total follow‐up visits. A total of 37 patients reported suspected treatment‐related AEs across 60 visits, with a median onset of 4 months after therapy initiation. Of these events, 48% were subjectively classified as mild, 38% as moderate, and 13% as severe. The most common AE categories were musculoskeletal complaints (24%), skin reactions (23%), and injection‐site reactions (13%). Less frequent events included fatigue, ophthalmic issues, weight changes, and gastrointestinal, neurological, or psychological symptoms. Despite eight events being subjectively rated as severe by patients, only two patients (0.9% of total cohort) opted to discontinue dupilumab in this cohort; instead, most opted to extend the dosing interval. CRSwNP‐related exacerbations on dupilumab therapy were reported in 19% of patients, whereas 26% of patients reported doing so well that they ceased all topical therapy organically (Table 2).

**TABLE 2:** Adverse events (AE) and exacerbations during dupilumab treatment.

Measure	*N*, % or range
Patients with ≥1 AE	16.5%
Total adverse events	60
Median time to first AE	3.9 months (range 0–45)

Severity of AEs	Percentage
Mild	48.3%
Moderate	38.3%
Severe	13.3%

Symptom	Percentage
Musculoskeletal	24.0%
Skin	23.1%
Injection site reaction	13.2%
Fatigue/malaise	9.9%
Ophthalmic	7.4%
Weight change	5.8%
Gastrointestinal	5.0%
Neurological	4.1%
Psychological	2.5%
Other	4.2%

Event	Percentage
Oral steroids (new use after baseline)	14.0%
Antibiotics (new use after baseline)	21.1%
Exacerbations (new after baseline)	19.0%
Stopped intranasal steroids	25.8%

*Note*: Percentages for severity and by system affected are calculated from total AE count. Median time to first AE reflects the earliest recorded AE per patient. For events related to exacerbations, percentages are based on the number of patients with a clearly recorded yes or no.

**TABLE 3:** Comparison of standard (every 2 weeks) versus extended dosing (>2 weeks) of dupilumab in clinical outcome parameters.

Outcome	Time bin (months)	Standard (*n* = 134), median [IQR]	Extended (*n* = 90), median [IQR]	*p* value
TPS	0–12	2.00 [0.00–4.00]	2.00 [0.00–4.00]	0.075
	12–24	0.00 [0.00–2.00]	0.00 [0.00–1.00]	0.533
	24–36	0.00 [0.00–3.00]	0.00 [0.00–0.00]	0.286
	36–48	0.00 [0.00–1.00]	0.00 [0.00–1.00]	0.629
	48–60	1.00 [0.50–2.50]	0.00 [0.00–1.00]	0.533
Sniffin’ sticks 16 identity test	0–12	9.00 [5.00–12.00]	10.00 [5.00–12.00]	0.363
	12–24	11.00 [8.00–13.00]	11.00 [8.00–12.00]	0.963
	24–36	11.00 [8.00–12.00]	11.00 [8.00–12.00]	0.957
	36–48	9.50 [7.25–11.00]	10.00 [7.00–12.00]	0.629
	48–60	7.50 [5.50–10.25]	9.00 [6.00–12.50]	0.802
SNOT‐22	0–12	23.00 [9.00–50.50]	15.00 [7.00–35.00]	0.025
	12–24	10.00 [4.00–17.00]	10.00 [3.50–22.00]	0.960
	24–36	8.00 [3.00–28.50]	7.00 [2.25–14.75]	0.624
	36–48	7.50 [2.69–15.00]	8.00 [3.00–13.00]	0.987
	48–60	11.00 [9.00–14.00]	5.00 [2.50–8.76]	0.533
ACT	0–12	22.00 [19.00–25.00]	24.00 [22.00–25.00]	0.005
	12–24	24.00 [23.00–25.00]	25.00 [24.00–25.00]	0.533
	24–36	24.00 [18.75–25.00]	25.00 [24.00–25.00]	0.533
	36–48	24.50 [24.00–25.00]	25.00 [25.00–25.00]	0.286
	48–60	24.00 [20.00–24.50]	25.00 [24.25–25.00]	0.629
AQLQ	0–12	89.00 [70.50–99.00]	97.00 [86.00–102.00]	<0.001
	12–24	99.50 [85.54–02.21]	99.00 [92.50–103.00]	0.629
	24–36	97.75 [89.00–100.75]	102.00 [98.00–105.00]	0.112
	36–48	101.00 [99.00–103.75]	103.00 [98.00–105.00]	0.533
	48–60	94.00 [80.50–98.00]	103.00 [97.00–104.25]	0.533
Allergy VAS	0–12	7.90 [5.50–9.10]	8.10 [5.90–9.35]	0.533
	12–24	9.20 [7.40–9.65]	9.00 [7.90–9.60]	0.802
	24–36	9.00 [6.08–9.83]	8.90 [7.05–9.65]	0.963
	36–48	9.65 [5.80–9.88]	9.70 [8.90–10.00]	0.629
	48–60	10.00 [9.65–10.00]	9.40 [6.95–9.70]	0.802

*Notes*: Values are median with [interquartile range] within each follow‐up bin for standard versus extended dosing groups. Between‐group differences were tested using the Mann–Whitney *U* test.

Abbreviations: ACT, asthma control test; AQLQ, asthma quality of life questionnaire; SNOT‐22, 22 item sinonasal outcome test; TPS, total polyp score; VAS, visual analogue scale.

**TABLE 4:** Comparison of standard (every 2 weeks) versus extended (>2 weeks) dosing of dupilumab in laboratory outcome parameters.

Outcome	Time bin (months)	Standard (*n* = 134), median [IQR]	Extended (*n* = 90), median [IQR]	*p* value
Total IgE (kU/L)	0–12	71.30 [26.65–165.00]	49.20 [23.25–153.50]	0.4355
	12–24	16.40 [10.50–51.70]	21.70 [9.82–67.40]	0.7024
	24–36	9.37 [7.26–14.40]	20.30 [10.30–61.50]	0.0969
	36–48	8.96 [7.88–13.80]	16.90 [8.28–43.10]	0.2773
	48–60	16.05 [4.24–31.03]	17.00 [8.88–39.02]	0.8849
ECP (µg/L)	0–12	55.00 [28.80–95.70]	52.85 [29.20–91.97]	0.9629
	12–24	37.65 [22.77–77.10]	43.20 [25.40–73.90]	0.8849
	24–36	35.70 [17.00–59.70]	35.40 [20.70–55.20]	0.8871
	36–48	50.40 [35.15–76.75]	40.35 [20.75–62.23]	0.7024
	48–60	20.25 [19.38–48.58]	21.85 [18.68–30.28]	1.0000
Absolute eosinophil count (×10^9^/L)	0–12	0.30 [0.20–0.60]	0.30 [0.20–0.50]	0.8871
	12–24	0.30 [0.20–0.50]	0.30 [0.18–0.50]	0.8871
	24–36	0.25 [0.10–0.50]	0.30 [0.15–0.50]	0.8871
	36–48	0.40 [0.20–0.70]	0.30 [0.20–0.60]	0.8849
	48–60	0.25 [0.20–0.45]	0.25 [0.12–0.30]	0.8849

*Notes*: Values are median [IQR] within each follow‐up bin for standard versus extended dosing groups. Between‐group differences were tested using the Mann–Whitney *U* test.

Abbreviations: ECP, eosinophil cationic protein; IgE, immunoglobin E.

### 3.4 Patients Who Fail on Dupilumab Therapy Have Lower Markers of Type 2 Inflammation

During follow‐up, 19 patients (8.5%) had a confirmed discontinuation of dupilumab therapy. Among these, 10 patients (4.5% of the total cohort) discontinued treatment due to insufficient clinical response. Two patients (0.9%) stopped therapy due to AEs, while seven patients (3.1%) discontinued for nonefficacy‐related reasons, including pregnancy (three patients), achieving complete disease control and declining further therapy (two patients), or switching to an alternative biologic for asthma management (two patients). An additional 16 patients (7.1%) were lost to follow‐up and their treatment status is unknown (Table S1). Among patients who discontinued dupilumab due to insufficient clinical response, the median time to therapy failure was 119 days (IQR 84–177).

As a further exploratory analysis, baseline characteristics of patients who discontinued dupilumab due to insufficient clinical response were compared descriptively with those of patients who continued therapy. Patients who discontinued for nonefficacy reasons, AEs, or were lost to follow‐up were excluded from this analysis. Given the small number of confirmed therapy failures, all analyses are considered exploratory. Patients with therapy failure demonstrated lower baseline Type 2 inflammatory signals compared with those who continued treatment. None of the patients who discontinued due to insufficient response had N‐ERD, compared with 39.2% of patients who continued therapy (*p* = 0.014). In addition, patients with therapy failure had numerically lower baseline total IgE levels (median 26.7 vs. 86.2 kU/L; *p* = 0.054) and lower and absolute blood eosinophil counts (median 0.30 vs. 0.40 × 10^9^/L; *p* = 0.08), which almost reached significance as well as lower serum ECP concentrations (median 27.9 vs. 54.2 µg/L; *p* = 0.20). No differences were observed for age, sex, asthma status, smoking status, or prior sinus surgery (Table S2).

### 3.5 Patients Who Increase Their Dosing Interval Do Not Lose Benefit From Dupilumab

**FIGURE 3:**
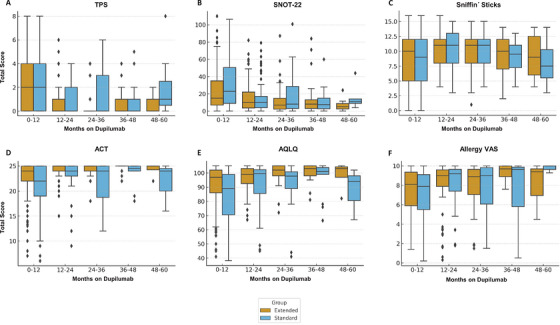
Clinical outcomes by dupilumab dosing interval. Boxplots show distributions for patients remaining on standard 2‐weekly dosing (blue) versus those on extended intervals (>2 weeks, orange), for (A) total polyp score (TPS), (B) sinonasal outcome test 22 (SNOT‐22), (C) Sniffin’ sticks 16 identity test, (D) asthma control test (ACT), (E) asthma quality of life questionnaire (AQLQ), and (F) burden of allergic symptoms represented by allergic visual analogue scale (VAS). Patients are stratified by follow‐up time (0–12, 12–24, 24–36, 36–48, 48–60 months). Boxes represent the interquartile range (IQR) with median line; whiskers extend to 1.5× IQR.

**FIGURE 4:**
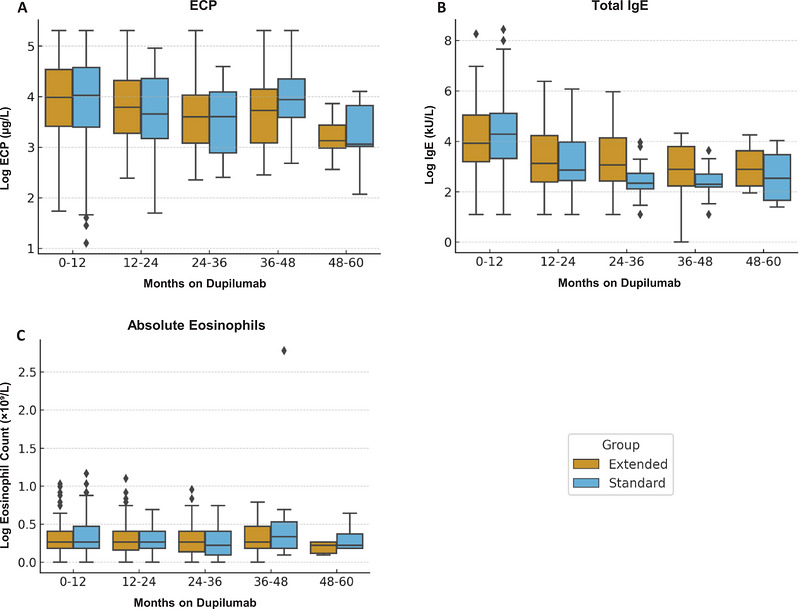
Laboratory outcomes by dupilumab dosing interval. Boxplots show distributions for patients remaining on standard 2‐weekly dosing (blue) versus those on extended intervals (>2 weeks, orange), for (A) eosinophil cationic protein (ECP), (B) total IgE, and (C) absolute eosinophils. Patients are stratified by follow‐up time (0–12, 12–24, 24–36, 36–48, 48–60 months). Boxes represent the interquartile range with median line; whiskers extend to 1.5× IQR.

40.1% (90 out of 224) of patients opted to increase their dosing interval after initial disease control. Among these, 46% extended to a 3‐week interval, 39% to a 4‐week interval, and 15% extended beyond 4 weeks. The median time to first dosing interval extension was 781 days (IQR 488–1141 days). Disease stability was defined as endoscopically as no worsening of disease on examination after clear response to treatment and the median time after this was achieved to dosing interval extension was 465 days (IQR 243–937 days). Baseline characteristics were similar between standard and extended dosing groups (Table S3). In the first year after therapy initiation, patients who increased their interval had significantly better clinical outcomes than those who remained on a 2‐week schedule. Specifically, ACT scores were higher in the extended group (median 24.00 [IQR 22.00–25.00] compared with the standard group (22.00 [IQR 19.00–25.00]; *p* = 0.0052). AQLQ scores were also higher in the extended group (97.00 [IQR 86.00–102.00] vs. 89.00 [IQR 70.50–99.00]; *p* < 0.0006), and SNOT‐22 scores were lower, indicating better symptom control (15.00 [IQR 7.00–35.00] vs. 23.00 [IQR 9.00–50.50]; *p* = 0.0256). These differences likely reflect that patients who opted to increase dosing intervals were initially well controlled and these group differences then vanished over time, with no sustained differences observed beyond the first year. No other outcome measures or changes in biomarkers over time (Figures 3 and 4 and Tables 3 and 4) differed significantly between the groups at later time points, suggesting that interval extension does not compromise long‐term effectiveness. Among patients who achieved dosing interval extension, a small subset (*n* = 10) subsequently required re‐intensification to a shorter dosing interval. Four of these went back down to 2 weekly dosing whereas the other six maintained some form of extended dosing interval typically returning to 4 weekly dosing after a trial of a more extended dosing interval. The most stable dosing interval was 3–4 weeks accounting for 76% of stable extensions. The median time from first interval extension to subsequent reduction was 364 days (IQR 275–409). Baseline characteristics of patients who needed to reduce again after extending were compared with stable extenders and overall, demographic, clinical, and biomarker parameters were broadly similar between groups. However, the presence of N‐ERD was more frequent among patients who later required dosing interval reduction compared with stable extenders (80.0 vs. 38.0%, *p* = 0.020). Given the small number of patients requiring interval reduction, this finding should be interpreted as exploratory (Table S4).

### 3.6 AEs Decrease After Dosing Interval Extension

To explore whether AEs influenced patients’ decisions to extend their dosing interval, we compared AE rates across all visits between groups. Patients in the extended group reported AEs more frequently overall (43.3 vs. 21.6%, *p* = 0.0007), although rates of severe AEs were similar (3.3 vs. 3.0%, *p* = 1.0000) (Table S5). Importantly, when considering only AEs that occurred after the dosing interval was extended, the incidence dropped to levels comparable to those seen in the standard group (21.1 vs. 21.6%, *p* = 1.0000) (Table S5). Among the 29 patients in the extended group who reported AEs before increasing their interval, only 10 continued to report events afterward. Of the three patients who had experienced what they rated as severe AEs prior to extension, none reported further severe events afterward (Table S6). The median number of AEs per patient decreased significantly from 1 [IQR 1–2] before interval extension to 0 [IQR 0–1] afterward (*p* = 0.0163) (Table S7). These findings suggest that increasing the injection interval may alleviate treatment‐related side effects in a subset of patients without compromising efficacy.

### 3.7 Prior Sinus Surgery is Associated With Durable Dupilumab Dosing Interval Extension

Baseline predictors of durable dosing interval extension were examined using univariable and multivariable analyses. In the whole extended dosing cohort, no baseline characteristics differed significantly between groups (Table S3). However, in a sensitivity analysis excluding patients who initially extended dosing but subsequently required interval reduction, prior sinus surgery was significantly associated with durable dosing interval extension, both in univariable analysis (42.0 vs. 27.6%, *p* = 0.039) and after multivariable adjustment (OR 2.13, 95% CI 1.07–4.27; *p* = 0.032). No other baseline clinical characteristic was significantly associated with durable dosing interval extension (Tables S8 and S9).

## 4 Discussion

This real‐word study of CRSwNP patients including a large cohort of N‐ERD patients treated with dupilumab demonstrated rapid and significant early clinical improvements in olfactory function (SSIT 16), polyp burden (TPS), asthma (ACT, AQLQ), allergy (VAS), and disease specific QoL (SNOT‐22) within the first 6 months. After this period, the parameters stabilized and showed minimal additional changes. Dupilumab was well tolerated, with 40% of patients choosing to extend their dosing interval after achieving initial disease control, without loss of benefit. AEs were generally low, predominantly mild or moderate, and appeared to decrease further following dosing interval extension.

Consistent with our findings, multiple other studies of CRSwNP patients on dupilumab therapy report a rapid onset of favorable outcomes. The SINUS‐24 and SINUS‐52 Phase 3 trials observed improvements in nasal polyp scores, nasal congestion, and SNOT‐22 scores as early as 4–8 weeks after treatment initiation, with peak responses typically occurring by 24 weeks and sustained through 52 weeks in SINUS‐52. [9, 18]. Real‐world studies have confirmed that these rapid responses to treatment are also achieved in routine clinical practice [11, 12, 13, 19]. The largest such cohort to date, including 925 CRSwNP patients, reported the greatest improvements in polyp scores, smell, and SNOT‐22 by 12 months, with effects persisting through 2 years [19]. Studies beyond 2 years are much more limited, but there are a few that agree with our findings showing improvements in nasal polyps, olfaction, nasal obstruction, and QoL measures are maintained at 30, 36, and 60 months [11, 12, 13]. The total sample sizes here are, however, small and have therefore lacked the statistical power to confidently generalize their findings.

Our analysis also demonstrated substantial overall improvements in both AQLQ and ACT scores, with the majority of changes occurring within the first 6 months and minimal additional changes thereafter. In line with these findings, real‐world data of patients with severe asthma have shown that dupilumab provides sustained improvements in asthma control, lung function, and biomarker profiles, including in those with prior biologic therapy. While not all patients achieve full symptom remission, early treatment response appears to be a strong predictor of long‐term success [20]. A similar pattern is observed in patients with CRSwNP and coexisting N‐ERD, who typically present with a higher baseline disease burden. In these patients, dupilumab elicits significant clinical benefit, improving polyp size and olfactory function and reducing Type 2 inflammatory biomarkers, despite starting from worse disease status compared with non‐N‐ERD patients. [21]. These observations underscore the consistent efficacy of dupilumab across patients with different Type 2 airway diseases.

The trajectories we observed with the Type 2 biomarkers, IgE, ECP, and AEC, are also consistent with previously published data [22, 23]. We noted a late secondary rise in AEC and ECP. These findings could be artefactual due to reduced datapoints at later timepoints but have also been noticed in atopic dermatitis and were importantly not associated with any increase in adverse outcomes [24]. In patients with CRSwNP and coexisting allergic rhinitis, treatment with dupilumab has also been shown to effectively suppress both total and allergen‐specific IgE levels in serum as well as in the nasal mucosal lining. These patients demonstrated a marked improvement in overall respiratory allergic burden, with reduced frequency and severity of allergic symptoms and decreased dependence on concomitant medications such as antihistamines and corticosteroids [25]. Consistent with these findings, our data demonstrate that this improvement is sustained over time, with allergy VAS scores showing a significant increase at 3 and 6 months, followed by a durable long‐term improvement thereafter.

Another area of active research is uncovering patients who are nonresponders to dupilumab therapy. Our exploratory analysis of patients who failed therapy found that they were characterized by approaching significant lower baseline total IgE and eosinophil counts and a significant total absence of comorbid N‐ERD. This suggests that nonresponse may be linked to a “pauci‐immune” or non‐Type 2 dominant endotype, such as neutrophilic or mixed (Type 1/Type 17) inflammation, which remains largely independent of the IL‐4 and IL‐13 signaling inhibited by dupilumab and has been shown to respond worse to biologic therapy [26]. While posthoc analyses from the SINUS‐24 and SINUS‐52 trials suggest that dupilumab remains superior to placebo even below typical biomarker thresholds, the interaction analysis showed that the most pronounced objective benefits were reserved for patients with baseline blood eosinophils >150 cells/µL [27]. Other real‐world studies support our observation that low baseline Type 2 signals correlate with clinical failure; specifically, pretreatment blood eosinophil counts have been found to positively correlate with nasal polyp score reduction [28], implying that patients with the lowest counts may be the most refractory to biological blockade.

Dupilumab demonstrated a favorable safety profile, with low rates of severe AEs and generally good tolerability. This aligns with findings from large cohorts, including a 5‐year real‐world study of 1286 AD patients, where most AEs were mild‐to‐moderate and only 7.6% of patients discontinued treatment due to side effects [29]. In our cohort, only two patients discontinued treatment due to AEs, and many chose to extend their dosing intervals. Interval prolongation was associated with fewer AEs, improved symptom control, and better QoL over the first year, without compromising long‐term outcomes. Existing real‐world studies have initiated dosing de‐escalation for several reasons, including persistent blood eosinophilia, management of nonsevere AEs, and patient‐driven requests after achieving stable disease. It has also been emphasized that interval extension can be considered a patient‐specific, individualized treatment adjustment, implemented only after substantial clinical improvement, absence of systemic corticosteroid use, and adequate control of any coexisting asthma [30, 31]. Real‐world data confirm that extending intervals from 2 to 4 weeks, or even up to 8 weeks, maintains efficacy and can reduce or fully resolve side effects, suggesting improved tolerability over time [30, 31]. However, the exact mechanisms underlying this biological adaptation remain to be understood. One possible explanation, as discussed in the literature, is the sustained IL‐4Rα saturation occurring from high dupilumab concentrations in serum, combined with the abundant IL‐4Rα presence, allowing continued disease suppression even with longer dosing intervals [31]. Furthermore how far dosing intervals can be pushed and who is likely to remain in remission on “off label” dupilumab dosing regimens is an important area under investigation. Our study identified prior sinus surgery as a significant independent predictor of durable dosing interval extension (OR 2.13). This finding aligns with emerging concepts of sinus surgery, and that patients who have “complete” sinus surgery do better on biologic therapy due to debulking of diseased and inflamed mucosa helps downregulate the inflammatory drive, lowering the biological threshold required to maintain clinical quiescence with less frequent dosing [32, 33]. Conversely, we interestingly found that although N‐ERD patients respond well to dupilumab, in agreement with others [34], they were the most consistent group to fail stable extensions, which aligns with the established literature that the high baseline inflammatory burden and aggressive nature of N‐ERD likely exceed other CRSwNP phenotypes and thereby necessitate more frequent dosing to prevent rapid eosinophilic recurrence and maintain sustained suppression [35].

Finally, the question of whether dupilumab therapy can be safely discontinued after achieving sustained clinical remission has also been explored in real‐world settings, partly driven by the high long‐term treatment costs of biologics. Interestingly, a Japanese study including a small cohort of atopic dermatitis patients treated with dupilumab found that, after an attempt to stop therapy, over half of the patients remained in symptom remission for roughly 40 weeks on average [36]. However, continuous disease suppression cannot be guaranteed, and ongoing treatment may still be necessary for some patients, as evidenced by one real‐world case of significant symptom worsening 3 months after treatment cessation [23].

Overall, these findings highlight that interval extension is likely to be a viable strategy for patients achieving early clinical improvement, potentially reducing treatment burden, cost, and the cumulative risk of AEs. In addition, for patients experiencing AEs on standard dosing despite evidence of treatment response, extending the dosing interval may represent a pragmatic option to reduce the frequency of subsequent AEs without necessarily compromising treatment benefit. From a patient's perspective, longer dosing intervals may also improve adherence to treatment and QoL, by decreasing the frequency of injections and clinical visits. However, selection bias should be considered, as in our study interval prolongation was typically chosen by patients who showed an early good response to therapy. By longitudinally tracking AEs in a real‐world setting, our study adds novel insight into the timing, persistence, and evolution of mild‐to‐moderate events, highlighting both the overall safety of dupilumab therapy and the potential for AEs to decline over time, particularly with extended dosing intervals.

This study offers several key strengths, providing important insights into the use of dupilumab in everyday clinical practice. Its relatively large cohort including a substantial number of N‐ERD patients and long‐term follow‐up enhances the robustness of the findings despite the limitations of its retrospective nature. Similarly, although comparisons between dosing interval groups were nonrandomized, the inclusion of a heterogeneous real‐world population with variable follow‐up intervals increases the generalizability of the results. Potential selection and reporting biases are mitigated by comprehensive longitudinal monitoring of PROMs and biomarker data. Nevertheless, although longitudinal mixed‐effects modeling allowed all available follow‐up data to contribute to estimate long‐term trajectories, only a small number of patients had observed follow‐up beyond 4 years. As such, estimates at later time points may be influenced by attrition and responder selection bias and should be interpreted cautiously. Finally, although the observational nature limits definitive conclusions about causality, the study still offers valuable real‐world evidence on the safety and effectiveness of dupilumab.

Our findings have several implications for clinical practice and future research. Stratified treatment approaches based on baseline patient characteristics may help optimize outcomes and identify those most suitable for dupilumab therapy and potential interval extension. Prospective studies are needed to establish safe thresholds for extending dosing intervals, while long‐term immune monitoring could further guide personalized biologic dosing strategies. Last, future research should assess the cost‐effectiveness of individualized biologic regimens in patients with CRSwNP to support evidence‐based, patient‐centered care.

## 5 Conclusion

Dupilumab provides sustained long‐term benefits across both clinical and biomarker measures in patients with CRSwNP and is tolerable in long term. Baseline patient characteristics may help to identify those most likely to reach meaningful improvement, supporting a more personalized treatment approach. Patient‐led extension of dosing intervals appears safe and effective in selected individuals, offering potential advantages in reducing treatment burden and cumulative AEs. Overall, these findings provide real‐world validation of dupilumab's role in the management of chronic upper airway disease and underscore the value of individualized, evidence‐based care.

## Author Contributions

S.S., N.J.C., and J.E.D. designed the study. S.S., A.T., N.J.C., F.C.F., N.F.M., K.G., T.J.B., D.L., and M.P. performed all data collection. N.J.C., D.P., S.S., and J.E.D. interpreted the data and designed the figures. All authors wrote and critically revised the manuscript together.

## Funding

M.Z. is funded by Austrian Science Fund Grant KLP4891723, No other formal funding was received for the study.

## Ethics Statement

Retrospective analysis of this patient population was approved by the Ethical Committee at the Medical University of Vienna (EK: 1340/2025).

## Conflicts of Interest

JED served as a speaker and/or consultant and/or advisory board member for Sanofi, AstraZeneca, and GSK. JED is an investigator for Sanofi, GSK, and AstraZeneca (grants paid to institution). SS served as a speaker and advisory board member for Sanofi, AstraZeneca, and GSK. SS is an investigator for Sanofi, AstraZeneca, and GSK (grants paid to institution). The other authors declare no conflicts of interest.

## Consent

The authors have nothing to report.

## Supporting information




**Supporting Table 1**: Reasons for dupilumab discontinuation or patients lost to follow‐up during treatment. 1 patient due to severe conjunctivitis and 1 due to severe joint pain. † 3 patients due to pregnancy, 2 patients wished to trial stopping therapy after achieving disease control and did not return thereafter, 2 patients were switched to benralizumab by their respiratory team due to their co‐morbid asthma.
**Supporting Table 2**: Baseline Characteristics comparing patients who failed dupilumab therapy to those treated successfully: values were compared among baseline visits if available. Continuous variables are summarized as median and [interquartile range (IQR)] and compared using Mann–Whitney *U* test; categorical variables are summarized as *n* and percentage (%) and compared with chi‐square or Fisher's exact tests as appropriate. For biomarkers (ECP, IgE, and absolute eosinophils), data were not available at all time points for all patients. *Abbreviations*: N‐ERD = nonsteroidal anti‐inflammatory drug exacerbated respiratory disease, IgE = immunoglobin E, ECP = eosinophil cationic protein.
**Supporting Table 3**: Baseline characteristics of all patients who extended dupilumab dosing (>2 weeks) versus standard (every 2 weeks) dosing. Values were compared among baseline visits if available. Continuous variables are summarized as median and [interquartile range (IQR)] and compared using Mann–Whitney *U* test; categorical variables are summarized as *n* and percentage (%) and compared with chi‐square or Fisher's exact tests as appropriate. For biomarkers (ECP, IgE, and absolute eosinophils), data were not available at all time points for all patients. *Abbreviations*: N‐ERD = nonsteroidal anti‐inflammatory drug exacerbated respiratory disease, IgE = immunoglobin E, ECP = eosinophil cationic protein.
**Supporting Table 4**: Baseline characteristics comparing stable extenders of dupilumab dosing versus later reducers: values were compared among baseline visits if available. Continuous variables are summarized as median and [interquartile range (IQR)] and compared using Mann–Whitney *U* test; categorical variables are summarized as *n* and percentage (%) and compared with chi‐square or Fisher's exact tests as appropriate. For biomarkers (ECP, IgE, and absolute eosinophils), data were not available at all time points for all patients. *Abbreviations*: N‐ERD = nonsteroidal anti‐inflammatory drug exacerbated respiratory disease, IgE = immunoglobin E, ECP = eosinophil cationic protein.
**Supporting Table 5**: Adverse reactions (AE) by dosing interval group: standard (every 2 weeks) vs extended dosing (>2 weeks). Outcomes were compared with chi‐square or Fisher's exact test.
**Supporting Table 6**: Timing of adverse events (AE) relative to dupilumab dosing interval increase. The timing of adverse events relative to the first recorded dosing interval increase among patients who ever increased beyond 2 weeks. Values are presented as number of patients.
**Supporting Table 7**: Adverse events (AE) before versus after dupilumab dosing interval increase. Data are summarized as median per patient with interquartile range (IQR). The Wilcoxon signed‐rank test was used for paired comparison.
**Supporting Table 8**: Baseline predictors of subsequent dosing‐interval extension: standard (every 2 weeks) versus extended dosing (>2 weeks). Patients who later reduced their dosing interval after extending, were excluded from this analysis (*n* = 10). Values were compared among baseline visits if available. Continuous variables are summarized as median and [interquartile range (IQR)] and compared using Mann–Whitney *U* test; categorical variables are summarized as *n* and percentage (%) and compared with chi‐square or Fisher's exact tests as appropriate. For biomarkers (ECP, IgE, and absolute eosinophils), data were not available at all time points for all patients. *Abbreviations*: N‐ERD = nonsteroidal anti‐inflammatory drug exacerbated respiratory disease, IgE = immunoglobin E, ECP = eosinophil cationic protein.
**Supporting Table 9**: Multivariable logistic regression of baseline predictors of subsequent dosing‐interval extension: standard (every 2 weeks) versus stable extended dosing (>2 weeks). Patients who later reduced their dosing interval after extending, were excluded from this analysis (*n* = 10). Odds ratios (ORs) are presented with 95% confidence intervals (CIs). *Abbreviation*: N‐ERD = nonsteroidal anti‐inflammatory drug exacerbated respiratory disease.

## Data Availability

The data that support the findings of this study are archived at the Medical University of Vienna but restrictions apply to the availability of these data and so they are not publicly available. Data may however be made available at reasonable request to the authors and only with permission of the Medical University of Vienna.

## References

[1] H. K. Min , S. Lee , S. Kim , et al., “Global Incidence and Prevalence of Chronic Rhinosinusitis: A Systematic Review,” Clinical and Experimental Allergy 55, no. 1 (2025): 52–66. 39506931 10.1111/cea.14592

[2] N. J. Campion , R. Kohler , R. Ristl , S. Villazala‐Merino , J. Eckl‐Dorna , and V. Niederberger‐Leppin , “Prevalence and Symptom Burden of Nasal Polyps in a Large Austrian Population,” The Journal of Allergy and Clinical Immunology: In Practice 9, no. 11 (2021): 4117–4129 e2. 34265447 10.1016/j.jaip.2021.06.037

[3] R. E. Gliklich and R. Metson , “The Health Impact of Chronic Sinusitis in Patients Seeking Otolaryngologic Care,” Otolaryngology ‐ Head and Neck Surgery 113, no. 1 (1995): 104–109. 7603703 10.1016/S0194-59989570152-4

[4] E. S. Lourijsen and W. J. Fokkens , “Reitsma S. Direct and Indirect Costs of Adult Patients With Chronic Rhinosinusitis With Nasal Polyps,” Rhinology 58, no. 3 (2020): 213–217. 32415826 10.4193/Rhin19.468

[5] I. Ogulur , Y. Mitamura , D. Yazici , et al., “Type 2 Immunity in Allergic Diseases,” Cellular & Molecular Immunology 22, no. 3 (2025): 211–242. 39962262 10.1038/s41423-025-01261-2 PMC11868591

[6] W. J. Fokkens , V. J. Lund , C. Hopkins , et al., “European Position Paper on Rhinosinusitis and Nasal Polyps 2020,” Rhinology 58, no. Suppl S29 (2020): 1–464. 10.4193/Rhin20.600 32077450

[7] B. J. Lipworth , R. Greig , R. Chan , and C. R. Kuo , “Reappraisal of Biologic Efficacy From Phase 3 Trials in Refractory Chronic Rhinosinusitis and Nasal Polyps,” The Journal of Allergy and Clinical Immunology: In Practice 13, no. 8 (2025): 1943–1951. 40340024 10.1016/j.jaip.2025.04.043

[8] European Medicines Agency: EMA/H/C/004390 ‐ Dupixent (dupilumab) Summary of Product Characteristics, European Medicines Agency, https://www.ema.europa.eu/en/documents/product‐information/dupixent‐epar‐product‐information_en.pdf, updated April 2025.

[9] C. Bachert , J. K. Han , M. Desrosiers , et al., “Efficacy and Safety of dupilumab in Patients With Severe Chronic Rhinosinusitis With Nasal Polyps (LIBERTY NP SINUS‐24 and LIBERTY NP SINUS‐52): Results From Two Multicentre, Randomised, Double‐blind, Placebo‐controlled, Parallel‐group Phase 3 Trials,” Lancet 394, no. 10209 (2019): 1638–1650. 31543428 10.1016/S0140-6736(19)31881-1

[10] M. Rodriguez‐Iglesias , C. Calvo‐Henríquez , and D. Martin‐Jimenez , “Effect of Dupilumab in CRSwNP Sinonasal Outcomes From Real Life Studies: A Systematic Review With Meta‐analysis,” Current Allergy and Asthma Reports 25, no. 1 (2025): 13. 39907855 10.1007/s11882-025-01192-y PMC11799128

[11] G. L. Fadda , C. Rustichelli , S. Soccal , et al., “Dupilumab in the Treatment of Severe Uncontrolled Chronic Rhinosinusitis With Nasal Polyps (CRSwNP) and Comorbid Asthma‐A Multidisciplinary Monocentric Real‐Life Study,” Biomedicines 13, no. 2 (2025): 501. 40002914 10.3390/biomedicines13020501 PMC11853246

[12] S. J. Kilty and A. Lasso , “Canadian Real‐World Study Long‐Term Clinical Results Using Dupilumab for Chronic Rhinosinusitis with Polyps,” Journal of Otolaryngology ‐ Head & Neck Surgery 53 (2024): 19160216241278659. 39345032 10.1177/19160216241278659 PMC11450752

[13] R. Book , A. Lazutkin , and R. Eliashar , “Long‐Term Real‐World Outcomes and Insights of Biologic Therapies in Chronic Rhinosinusitis With Nasal Polyps,” International Journal of Molecular Sciences 26, no. 10 (2025): 4694. 40429838 10.3390/ijms26104694 PMC12112334

[14] B. Weissman , K. Shen , O. L. Flanagan , S. Chowdhury , and J. Sawires , “Comparing Medical and Surgical Management of Chronic Rhinosinusitis: A Systematic Review of Dupilumab and Endoscopic Sinus Surgery,” Cureus 17, no. 2 (2025): e79742. 40161127 10.7759/cureus.79742 PMC11954151

[15] S. Cai , S. Xu , Y. Zhao , and L. Zhang , “Efficacy and Safety of Biologics for Chronic Rhinosinusitis with Nasal Polyps: A Meta‐Analysis of Real‐World Evidence,” Allergy 80, no. 5 (2025): 1256–1270. 39985317 10.1111/all.16499 PMC12105074

[16] J. W. Yunginger and G. J. Gleich , “Seasonal Changes in IgE Antibodies and Their Relationship to IgG Antibodies During Immunotherapy for Ragweed Hay Fever,” Journal of Clinical Investigation 52, no. 5 (1973): 1268–1275. 4735589 10.1172/JCI107294 PMC302383

[17] P. Gevaert , J. De Craemer , C. Bachert , et al., “European Academy of Allergy and Clinical Immunology Position Paper on Endoscopic Scoring of Nasal Polyposis,” Allergy 78, no. 4 (2023): 912–922. 36661567 10.1111/all.15650

[18] S. E. Lee , C. Hopkins , J. Mullol , et al., “Dupilumab Improves Health Related Quality of Life: Results From the Phase 3 SINUS Studies,” Allergy 77, no. 7 (2022): 2211–2221. 35034364 10.1111/all.15222 PMC9305228

[19] E. Corso , C. Montuori , C. Pipolo , et al., “Two‐Year Turning Point with Dupilumab in CRSwNP: Control, Remission, and Tapering Dosage,” Allergy (2025), n/a‐n/a. 10.1111/all.70032 40884177

[20] C. Mümmler , A. Lenoir , J. Götschke , et al., “Long‐term Outcomes of dupilumab Therapy in Severe Asthma: A Retrospective, Multicenter, Real‐world Study,” Journal of Allergy and Clinical Immunology: Global 4, no. 4 (2025): 100533. 40810090 10.1016/j.jacig.2025.100533 PMC12347924

[21] H. B. E. Elzinga , J. J. Otten , M. E. Cornet , et al., “Two‐Year Data of Tapered Dupilumab Shows High Effectiveness in Chronic Rhinosinusitis With Nasal Polyps with Nonsteroidal Anti‐inflammatory Drug‐Exacerbated Respiratory Disease,” Allergy 80, no. 6 (2025): 1737–1745. 40377347 10.1111/all.16579 PMC12186595

[22] A. G. Ledda , G. Costanzo , G. Sambugaro , et al., “Eosinophil Cationic Protein Variation in Patients With Asthma and CRSwNP Treated With Dupilumab,” Life (Basel) 13, no. 9 (2023): 1884. 37763288 10.3390/life13091884 PMC10532820

[23] S. O. Sarnoch , A. Pepić , L. Schmitz , B. Becker , C. Betz , and A. S. Hoffmann , “The Value of Biomarkers in the Therapy of CRSwNP With Biologicals‐a Long‐term Follow‐up of Dupilumab Therapy,” European Archives of Oto‐Rhino‐Laryngology 281, no. 9 (2024): 4789–4805. 38709320 10.1007/s00405-024-08574-4 PMC11393186

[24] A. Li , A. H. Musters , A. Hyseni , L. A. A. Gerbens , and P. I. Spuls , “Dupilumab‐associated (hyper)Eosinophilia in Patients With Atopic Dermatitis: A Single‐centre Cohort Study of the TREAT NL (TREatment of ATopic eczema, the Netherlands) Registry,” British Journal of Dermatology 191, no. 6 (2024): 1012–1013. 39005169 10.1093/bjd/ljae289

[25] N. J. Campion , A. Doralt , C. Lupinek , et al., “Dupilumab Reduces Symptom Burden in Allergic Rhinitis and Suppresses Allergen‐specific IgE Production,” Allergy 78, no. 6 (2023): 1687–1691. 36691369 10.1111/all.15653

[26] L. H. Png , L. Kalish , R. G. Campbell , et al., “Predictors of Persistent Disease in Biologic Treated Type 2 Diffuse/Eosinophilic Chronic Rhinosinusitis Undergoing Surgery,” International Forum of Allergy & Rhinology 14, no. 5 (2024): 909–918. 37805956 10.1002/alr.23282

[27] C. Bachert , P. Gevaert , B. Lipworth , et al., “Dupilumab Efficacy Across Serum IgE and Blood Eosinophil Levels in Chronic Rhinosinusitis With Nasal Polyposis,” Allergy 79, no. 10 (2024): 2858–2861. 38850227 10.1111/all.16189

[28] M. Habenbacher , U. Moser , A. Abaira , et al., “Investigation of Blood Count‐Based Inflammatory Biomarkers as Predictors of Response to Dupilumab Treatment in Patients With Chronic Rhinosinusitis With Nasal Polyps,” Pharmaceutics 16, no. 11 (2024): 1370. 39598494 10.3390/pharmaceutics16111370 PMC11597114

[29] C. M. Boesjes , E. Kamphuis , M. de Graaf , et al., “Long‐Term Effectiveness and Reasons for Discontinuation of Dupilumab in Patients with Atopic Dermatitis,” JAMA Dermatology 160, no. 10 (2024): 1044–1055. 39110432 10.1001/jamadermatol.2024.2517 PMC11307167

[30] E. De Corso , C. Montuori , and G. De Maio , “Dupilumab Monthly Dose De‐Escalation Maintains Efficacy in CRSwNP: A Two‐Year Real‐World Study,” Laryngoscope 135, no. 7 (2025): 2267–2274. 40152155 10.1002/lary.32162

[31] H. M. Appel , R. Lochbaum , T. K. Hoffmann , and J. Hahn , “Chronic Rhinosinusitis With Nasal Polyps‐extension of Dupilumab Treatment Intervals],” Hno 72, no. 7 (2024): 499–503. 38761229 10.1007/s00106-024-01487-y PMC11192668

[32] A. J. Yu , C. Phung , K. Kuppusamy , et al., “The Completeness of Surgery Index Predicts Success in CRSwNP with Asthma by SNOT‐22 and Asthma Control Test,” International Forum of Allergy & Rhinology (2025). 10.1002/alr.70043 41045292

[33] M. Alicandri‐Ciufelli , D. Marchioni , C. Pipolo , et al., “Influence of Prior Endoscopic Sinus Surgery Extent on Dupilumab Effectiveness in CRSwNP Patients,” Laryngoscope 134, no. 4 (2024): 1556–1563. 37632705 10.1002/lary.30983

[34] K. M. Buchheit , A. Sohail , J. Hacker , et al., “Rapid and Sustained Effect of dupilumab on Clinical and Mechanistic Outcomes in Aspirin‐exacerbated respiratory Disease,” Journal of Allergy and Clinical Immunology 150, no. 2 (2022): 415–424. 35460728 10.1016/j.jaci.2022.04.007 PMC9378638

[35] T. M. Laidlaw and J. A. Boyce , “Pathogenesis of Aspirin‐exacerbated respiratory Disease and Reactions,” Immunology and Allergy Clinics of North America 33, no. 2 (2013): 195–210. 23639708 10.1016/j.iac.2012.11.006 PMC3781366

[36] S. Miyamoto , Y. Imai , M. Natsuaki , K. Yamanishi , and N. Kanazawa , “Long‐term Remission of Atopic Dermatitis After Discontinuation of Dupilumab,” Acta Dermato‐Venereologica 102 (2022): adv00731. 35578819 10.2340/actadv.v102.295 PMC9609981

