# Supplementary material for: Dosing Interval Extension of Dupilumab in CRSwNP: Five‐Year Real World Outcomes
Source: Int Forum Allergy Rhinol. 2026 Mar 2;16(8):797–808. doi: 10.1002/alr.70125 (PMC13432586; doi:10.1002/alr.70125)
Supplement: Supplementary file 1 — Supporting Table 1: Reasons for dupilumab discontinuation or patients lost to follow‐up during treatment. 1 patient due to severe conjunctivitis and 1 due to severe joint pain. † 3 patients due to pregnancy, 2 patients wished to trial stopping therapy after achieving disease control and did not return thereafter, 2 patients were switched to benralizumab by their respiratory team due to their co‐morbid asthma. Supporting Table 2: Baseline Characteristics comparing patients who failed dupilumab therapy to those treated successfully: values were compared among baseline visits if available. Continuous variables are summarized as median and [interquartile range (IQR)] and compared using Mann–Whitney U test; categorical variables are summarized as n and percentage (%) and compared with chi‐square or Fisher's exact tests as appropriate. For biomarkers (ECP, IgE, and absolute eosinophils), data were not available at all time points for all patients. Abbreviations: N‐ERD = nonsteroidal anti‐inflammatory drug exacerbated respiratory disease, IgE = immunoglobin E, ECP = eosinophil cationic protein. Supporting Table 3: Baseline characteristics of all patients who extended dupilumab dosing (>2 weeks) versus standard (every 2 weeks) dosing. Values were compared among baseline visits if available. Continuous variables are summarized as median and [interquartile range (IQR)] and compared using Mann–Whitney U test; categorical variables are summarized as n and percentage (%) and compared with chi‐square or Fisher's exact tests as appropriate. For biomarkers (ECP, IgE, and absolute eosinophils), data were not available at all time points for all patients. Abbreviations: N‐ERD = nonsteroidal anti‐inflammatory drug exacerbated respiratory disease, IgE = immunoglobin E, ECP = eosinophil cationic protein. Supporting Table 4: Baseline characteristics comparing stable extenders of dupilumab dosing versus later reducers: values were compared among baseline visits if available. Continuous va [file ALR-16-797-s001.docx]

**Supplementary Material**

**Supplementary Tables**

| **Reason for discontinuation** | **Patients, n (%)** |
| --- | --- |
| **Insufficient response / therapy failure** | 10 (4.5%) |
| **Adverse events*** | 2 (0.9%) |
| **Lost to follow-up** | 16 (7.1%) |
| **Other**† | 7 (3.1%) |

**Supplementary Table 1: Reasons for dupilumab discontinuation or patients lost to follow-up during treatment.** * 1 patient due to severe conjunctivitis and 1 due to severe joint pain. † 3 patients due to pregnancy, 2 patients wished to trial stopping therapy after achieving disease control and didn’t return thereafter, 2 patients were switched to benralizumab by their respiratory team due to their co-morbid asthma.

| **Variable** | **Therapy failure (n = 10), Median [IQR] or %** | **Continued therapy (n = 189), Median [IQR] or %** | **p-value** |
| --- | --- | --- | --- |
| Age | 58.34 [35.29 – 62.39] | 48.09 [39.15 – 58.46] | 0.57 |
| Sex (male %) | 50.0% | 57.7% | 0.75 |
| ≥1 previous surgery (%) | 50.0% | 66.1% | 0.32 |
| Smoking (%) | 20.0% | 13.6% | 0.63 |
| Asthma (%) | 60.0% | 68.3% | 0.73 |
| N-ERD (%) | 0.0% | 39.2% | **0.014** |
| ECP (µg/L) | 27.90 [24.80 – 67.70] | 54.15 [29.88 – 83.65] | 0.20 |
| Total IgE (kU/L) | 26.70 [22.70 – 66.80] | 86.20 [36.60 – 232.25] | 0.054 |
| Absolute eosinophil count (×10⁹/L) | 0.30 [0.20 – 0.30] | 0.40 [0.20 – 0.50] | 0.08 |

**Supplementary Table 2: Baseline Characteristics Comparing Patients who Failed Dupilumab Therapy to those Treated Successfully:** Values were compared among baseline visits if available. Continuous variables are summarized as median and [interquartile range (IQR)] and compared using Mann–Whitney U; categorical variables are summarized as n and percentage (%) and compared with chi-square or Fisher's exact tests as appropriate. For biomarkers (ECP, IgE, and Absolute Eosinophils) data was not available at all time points for all patients. N-ERD = Non-steroidal anti-inflammatory drug exacerbated respiratory disease, IgE = Immunoglobin E, ECP = Eosinophil Cationic Protein

| **Variable** | **Standard (n = 134), Median [IQR] or %** | **Extended (n = 90), Median [IQR] or %** | **p-value** |
| --- | --- | --- | --- |
| **Age** | 49.11 [38.92 – 60.34] | 47.48 [37.11 – 54.22] | 0.490 |
| **Sex (male %)** | 57.5% | 56.7% | 1.000 |
| **≥1 previous surgery (%)** | 59.7% | 73.3% | 0.489 |
| **Smoking (%)** | 16.3% | 10.1% | 0.237 |
| **Asthma (%)** | 63.4% | 68.9% | 0.853 |
| **N-ERD (%)** | 32.8% | 42.2% | 0.488 |
| **ECP (µg/L)** | 51.20 [24.77 – 76.40] | 47.90 [29.40 – 78.60] | 1.000 |
| **Total IgE (kU/L)** | 108.50 [47.10 – 297.75] | 83.80 [40.55 – 326.50] | 0.651 |
| **Absolute Eosinophil Count (×10⁹/L)** | 0.30 [0.20 – 0.50] | 0.40 [0.20 – 0.50] | 0.712 |

**Supplementary Table 3: Baseline Characteristics of all Patients who Extended Dupilumab dosing (>2 weeks) vs Standard (every 2 weeks) Dosing.** Values were compared among baseline visits if available. Continuous variables are summarized as median and [interquartile range (IQR)] and compared using Mann–Whitney U; categorical variables are summarized as n and percentage (%) and compared with chi-square or Fisher's exact tests as appropriate. For biomarkers (ECP, IgE, and Absolute Eosinophils) data was not available at all time points for all patients. N-ERD = Non-steroidal anti-inflammatory drug exacerbated respiratory disease, IgE = Immunoglobin E, ECP = Eosinophil Cationic Protein

| **Variable** | **Reducers (n=10), Median [IQR] or %** | **Stable extenders (n=80), Median [IQR] or %** | **p value** |
| --- | --- | --- | --- |
| Age | 44.96 [35.42–49.03] | 47.89 [37.56–54.74] | 0.296 |
| Male sex (%) | 60.0% | 55.7% | 1.000 |
| ≥1 previous surgery (%) | 80.0% | 73.4% | 0.585 |
| Smoking (%) | 30.0% | 7.6% | 0.061 |
| Asthma (%) | 70.0% | 69.6% | 1.000 |
| **N-ERD (%)** | **80.0%** | **38.0%** | **0.020** |
| ECP (µg/L) | 57.15 [40.00–92.10] | 49.35 [28.07–86.30] | 0.368 |
| Total IgE (kU/L) | 75.00 [50.65–226.50] | 72.20 [31.00–235.50] | 0.613 |
| Absolute eosinophil count (×10⁹/L) | 0.35 [0.30–0.80] | 0.40 [0.20–0.50] | 0.524 |

**Supplementary Table 4: Baseline Characteristics Comparing Stable Extenders of Dupilumab Dosing vs Later Reducers:** Values were compared among baseline visits if available. Continuous variables are summarized as median and [interquartile range (IQR)] and compared using Mann–Whitney U; categorical variables are summarized as n and percentage (%) and compared with chi-square or Fisher's exact tests as appropriate. For biomarkers (ECP, IgE, and Absolute Eosinophils) data was not available at all time points for all patients. N-ERD = Non-steroidal anti-inflammatory drug exacerbated respiratory disease, IgE = Immunoglobin E, ECP = Eosinophil Cationic Protein

| **Adverse Event** | **Standard (%)** | **Extended (%)** | **p-value** |
| --- | --- | --- | --- |
| **Any AE** | 21.6% | 43.3% | 0.0007 |
| **Severe AE** | 3.0% | 3.3% | 1.0000 |

| **Adverse Event** | **Standard (%)** | **Extended after increase (%)** | **p-value** |
| --- | --- | --- | --- |
| **Any AE** | 21.6% | 21.1% | 1.0000 |
| **Severe AE** | 3.0% | 0.0% | 0.1508 |

**Supplementary Table 5: Adverse Reactions (AE) by Dosing Interval Group**: Standard (every 2 weeks) vs Extended Dosing (>2 weeks). Outcomes were compared with chi-square or Fisher’s exact test.

| **Event Category** | **Number of Patients** |
| --- | --- |
| **Any AE before first interval increase** | 29 |
| **Any AE after first interval increase** | 19 |
| **AE only before increase** | 19 |
| **AE only after increase** | 9 |
| **AE both before and after** | 10 |
| **Severe AE before increase** | 3 |
| **Severe AE after increase** | 0 |

**Supplementary Table 6. Timing of Adverse Events (AE) Relative to Dupilumab Dosing Interval Increase**. The timing of adverse events relative to the first recorded dosing interval increase among patients who ever increased beyond 2 weeks. Values are presented as number of patients.

| **Measure** | **Before increase (Median [IQR])** | **After increase (Median [IQR])** | **p-value** |
| --- | --- | --- | --- |
| **Number of AEs** | 1 [1 – 2] | 0 [0 – 1] | 0.0163 |

**Supplementary Table 7: Adverse Events (AE) Before vs After Dupilumab Dosing Interval Increase**. Data are summarized as median per patient with interquartile range (IQR). The Wilcoxon signed-rank test was used for paired comparison.

| **Baseline variable** | **% extended (Yes to Baseline Variable )** | **% extended (No to Baseline Variable)** | **p value** |
| --- | --- | --- | --- |
| Male sex | — | 36.9% | 1.000 |
| Smoking | 21.4% | 39.2% | 0.092 |
| Allergy | 38.3% | 35.4% | 0.673 |
| Asthma | 39.3% | 32.4% | 0.373 |
| N-ERD | 40.5% | 35.0% | 0.458 |
| ≥1 previous surgery | **42.0%** | **27.6%** | **0.039** |

**Supplementary Table 8: Baseline predictors of subsequent dosing-interval extension: Standard (every 2 weeks) vs Extended Dosing (>2 weeks).** Patients who later reduced their dosing interval after extending, were excluded from this analysis (n=10). Values were compared among baseline visits if available. Continuous variables are summarized as median and [interquartile range (IQR)] and compared using Mann–Whitney U; categorical variables are summarized as n and percentage (%) and compared with chi-square or Fisher's exact tests as appropriate. For biomarkers (ECP, IgE, and Absolute Eosinophils) data was not available at all time points for all patients. N-ERD = Non-steroidal anti-inflammatory drug exacerbated respiratory disease, IgE = Immunoglobin E, ECP = Eosinophil Cationic Protein

| **Variable** | **OR** | **95% CI** | **p value** |
| --- | --- | --- | --- |
| Age | 0.99 | 0.97–1.01 | 0.26 |
| Male sex | 0.92 | 0.51–1.66 | 0.79 |
| Asthma | 1.11 | 0.56–2.20 | 0.76 |
| N-ERD | 1.09 | 0.58–2.08 | 0.78 |
| Smoking | 0.38 | 0.14–1.04 | 0.059 |
| ≥1 previous surgery | **1.98** | **1.06–3.70** | **0.033** |

**Supplementary Table 9: Multivariable logistic regression of baseline predictors of subsequent dosing-interval extension: Standard (every 2 weeks) vs Stable Extended Dosing (>2 weeks).** Patients who later reduced their dosing interval after extending, were excluded from this analysis (n=10). Odds ratios (ORs) are presented with 95% confidence intervals (CIs). N-ERD = Non-steroidal anti-inflammatory drug exacerbated respiratory disease,
